# Caring for a friend or family member who has experienced suicidal behaviour: A systematic review and qualitative synthesis

**DOI:** 10.1111/papt.12449

**Published:** 2023-02-14

**Authors:** Paul Marshall, Keith Sansom, Glorianna Jagfeld, Steven Jones, Fiona Lobban

**Affiliations:** ^1^ Division of Health Research, Spectrum Centre for Mental Health Research Lancaster University Lancaster UK

**Keywords:** carers, families, qualitative synthesis, suicidal behaviour, systematic review

## Abstract

**Purpose:**

Friends and family members can be important sources of support for people who are or have been suicidal. This review aimed to synthesise qualitative evidence regarding carers' perspectives of supporting someone who has experienced suicidal behaviour.

**Methods:**

Five electronic databases (Web of Science, CINAHL, PsychINFO, MEDLINE, and SocINDEX) were searched from inception to May 2022. Eligible qualitative studies were published in English and investigated the caring experiences of friends or family members of people who had experienced suicidal behaviour, defined as any form of suicidal ideation and/or attempts. Studies focused on non‐suicidal self‐injury or suicide bereavement were excluded. Data from 19 eligible studies were analysed using thematic synthesis.

**Results:**

Initial carer reactions to suicidal behaviour included profound anxiety and intensive monitoring for signs of increased suicide risk amongst those they supported. Carers also reported significant challenges with understanding how to provide effective interpersonal support following suicidal crises. Professional support was perceived to be most effective when provided in a timely, interpersonally sensitive and safety‐focused manner. However, several studies detailed carers' difficulties accessing appropriate support and challenges managing their own distress.

**Conclusions:**

Carers face significant challenges with knowing how to respond to suicidal behaviour, where to find appropriate help, and how to manage their own distress. Future research should seek to investigate the effectiveness of easily accessible methods of information provision and support tailored for carers of people who have experienced suicidal behaviour.

**Practitioner points**

- Carers desire assistance from practitioners focused specifically on how to support their friends and family members in order to reduce suicide risk
- Practitioners should facilitate access to sources of support tailored to help carers manage the emotional impact of living alongside this risk
- Given the scale of carer support for people at risk of suicide, easily accessible, co‐designed resources tailored for managing suicidal behaviour in the community, such as online psychoeducation, represent a potentially valuable strategy for addressing these needs

## INTRODUCTION

Approximately 800,000 people die by suicide annually worldwide (World Health Organisation, 2019). Suicidal behaviour, including suicidal ideation or attempt (O'Connor & Nock, 2014), is many times more common, with cross‐national lifetime estimates of 9.2% and 2.7%, respectively (Nock et al., 2008). Social environments often play important roles in suicide‐related outcomes. For example, the integrated motivational‐volitional model of suicidal behaviour identifies perceived burdensomeness, thwarted belongingness and absence of social support as motivational moderators that strengthen links between feelings of entrapment and suicidal ideation (O'Connor & Kirtley, 2018). Conversely, higher social support is associated with lower risk of suicidal ideation and lifetime suicide attempt (Kleiman & Liu, 2013).

Providing care to a friend or family member with a healthcare need is an issue of international importance. Between 10% and 30% of adult populations in European countries self‐report participation in this form of care (Zigante, 2018). Challenges with identifying carers, including some not recognising that their supporting roles fall within this definition, likely lead to underestimation of the true scale of caregiving (National Institude for Health and Care Excellence, 2020). Caregiving in the context of suicidal behaviour is particularly complex and demanding. Carers experience difficulties interpreting suicidal intent and knowing when to communicate their concerns to others (Owens et al., 2011; Owen et al., 2012) report balancing the impulse to continuously guard their loved ones against the desire to promote their autonomy (Sun et al., 2009) and highlight contextual factors in recovery, including cultural beliefs about the meaning of suicide (Sun et al., 2008). Caring for a suicidal family member can also lead to poorer psychological well‐being and general health (Morgan et al., 2013) and increased caregiver burden (McDonell et al., 2003). However, prior research in this area highlights unresolved needs for effective carer‐inclusive interventions (Krysinska et al., 2021) and for healthcare professionals to build collaborative links with the families of people experiencing suicidal behaviour (Littlewood et al., 2019).

Considerable existing research provides insight into the lived experience of caregiving and suicidal behaviour. A review of 35 qualitative studies with parents highlighted how breakdown in communication with their suicidal children impeded caregiving and exacerbated hopelessness and shame within families (Simes et al., 2021). An earlier review of 44 qualitative studies, also with parents of children who had experienced suicidal behaviour, highlighted carers' experiences of low mood and disempowerment, a sense of relational distancing from their children, and difficulties implementing effective support strategies (Lachal et al., 2015). Juel et al. (2020) report a meta‐ethnographic analysis of 12 studies elucidating the process by which families seek to regain normality following experiences such as guilt, shame, powerlessness and anger in response to suicidal behaviour. Some carers experienced a sense of ‘feeling helpful’ in their relative's recovery, yet many felt powerless to bring about improvements in their family members' mental health, leaving them stuck with feelings of grief and loss. This literature reinforces the importance of equipping relatives with tools to manage suicidal behaviour and their own distress, as recommended by UK national (National Institute for Health and Care Excellence, 2019) and international healthcare guidance for suicide prevention (World Health Organisation, 2019).

Existing qualitative reviews have notable limitations. Some have applied study selection criteria including self‐injury regardless of intent (Juel et al., 2020; Simes et al., 2021). However, suicidal and nonsuicidal self‐injury can be distinct experiences with different triggers, functions, and relational implications (Cipriano et al., 2017). As per psychosocial theories of suicidal behaviour, absence of social integration or belonging, and negative perceptions of social support contribute to the desire to escape psychological pain through suicide (Mueller et al., 2021). However, nonsuicidal self‐injury, while potentially triggered by interpersonal difficulties (Edmondson et al., 2016), is often driven by a motivation to regulate the intensity of emotional experiences (Horne & Csipke, 2009). Focusing on studies of suicidal behaviour may therefore help to identify prominent and unique aspects of related caregiving experiences. Moreover, many studies focus on family carers, particularly parents (Lachal et al., 2015), yet other contacts including friends are important sources of support for people who are suicidal (Czyz et al., 2012; Giletta et al., 2017; Massing‐Schaffer et al., 2020). Indeed, UK clinical guidelines define a carer as someone who provides unpaid support to a family member, partner or friend with a health or social care need (National Institude for Health and Care Excellence, 2020). It is therefore important to broaden the scope of research in this area to align with this definition.

To date, qualitative systematic reviews have not typically synthesised carers' perspectives of providing support to friends or family members who have been affected by suicidal behaviour, including suicidal ideation and/or attempts. The aim of this review was therefore to address the question: ‘What are carers' experiences of supporting friends or family members who have experienced suicidal behaviour?’

## METHODS

### Design

Qualitative thematic synthesis as described by Thomas and Harden (2008) was selected as its realist ontological basis can aid in identifying meaningful recommendations for health research and practice (Booth et al., 2016). This review is reported with reference to PRISMA guidelines (Page et al., 2021).

### Search strategy

Search terms were developed in consultation with a specialist librarian at Lancaster University. Subject terms were added to improve search comprehensiveness and are detailed on PROSPERO: (https://www.crd.york.ac.uk/prospero/display_record.php?RecordID=226444).

Concepts were combined using the ‘AND’ Boolean operator:

Concept 1: suicid* OR self‐harm* OR self‐injur*.
Concept 2: carer* OR caregiv* OR famil* OR friend*.
Concept 3: qualitative OR mixed‐method* OR interview* OR focus‐group*.

Searches were applied to title, abstract, and keyword fields of PsycINFO, MEDLINE, CINAHL & SocINDEX via EBSCO and Web of Science, from database inception to 25 May 2022. Citation tracking was completed by hand‐searching reference sections and ‘cited by’ Google Scholar pages of eligible studies (Bakkalbasi et al., 2006).

### Study eligibility

Eligible studies:

- reported carers' experiences separately from other stakeholders. Carers were defined as those who provided unpaid support to a friend or family member, including partners (Roth et al., 2015).
- investigated caring for someone who had experienced suicidal behaviour, defined as any form of suicidal ideation and/or suicide attempts (O'Connor & Nock, 2014).
- used qualitative or mixed methods. Only the qualitative components of mixed‐methods studies were extracted for this review.
- were primary research articles published in English‐language peer‐reviewed journals.
- included carers aged 16 or over, reflecting the age at which carers are entitled to needs assessments and governmental assistance in the UK (National Health Service, 2021).

Ineligible studies:

- involved carers bereaved by suicide, as the experience of bereavement is substantially different from the focus of the research question.
- involved fewer than three participants to avoid reports based on a small number of participants disproportionately impacting the final analysis.

### Study selection

Database search results were deduplicated using Endnote (Bramer et al., 2016). Titles and abstracts were screened for eligibility by a primary and secondary reviewer using Rayyan (Ouzzani et al., 2016). Full texts of remaining studies were screened by the primary reviewer. A 20% of full texts were reviewed by the secondary reviewer to check for consistency in application of the screening procedure. Differences were resolved in open discussion between reviewers.

### Quality appraisal

Eligible studies were assessed by the first author using the Critical Appraisal Skills Programme (CASP) qualitative tool (CASP, 2018). Given the broad range of qualitative methodologies with varying definitions of research quality, this assessment was not used to determine a standard for inclusion or sensitivity analysis (Thomas & Harden, 2008), but rather to record the methodological transparency of relevant literature.

### Data extraction

Study characteristics (study aim, participant demographic characteristics, country, and methods of data collection and analysis) were extracted by the first author using a standardised data extraction form (Harris, 2011). Full results/findings sections (including author interpretation, participant quotes, tables, and figures) were copied verbatim into NVivo version 12 (QSR, 2018) for analysis. Only results detailing carers' experiences, and not those of other stakeholder such as professionals, were extracted for analysis.

### Data synthesis

Data were analysed using thematic synthesis (Thomas & Harden, 2008). Initial coding involved attaching labels to sections of text, briefly summarising their meaning. Codes were grouped by patterns of meaning into descriptive themes, which remained relatively close to the surface level meaning of the primary data. Through an iterative process of reviewing descriptive themes and their underlying data, these themes were grouped again by shared meaning and developed into analytic themes at a higher level of abstraction. Coding and the generation of initial candidate themes was completed by the first author. These candidate themes were reviewed, refined and finalised in discussion with the wider research team.

### Reflexivity

The authors adopted a critical realist philosophical position, which acknowledges that researchers' understandings of social reality are inevitably influenced by their idiosyncratic interpretive perspectives (Maxwell, 2012). We therefore sought to embed reflexivity, the practice of maintaining critical awareness of the influence of these perspectives on the research process, throughout the analysis (Finlay & Gough, 2008). The first author drew on the insider–outsider heuristic to facilitate reflexive thinking (Hellawell, 2006). This informed an initial reflexive statement, highlighting beliefs and expectations about the research topic. Ongoing reflexive writing throughout the review was used to remain cognizant of the shifting perspectives brought to the analysis and the lead author's relationship with the research topic. The study team included researchers with broad academic, clinical and lived experience of investigating and managing mental health difficulties in the context of caregiving and suicidal behaviour. Our aim was to draw on this experience to develop a nuanced analysis of carers' lived experiences through discussions and iterative written feedback.

## RESULTS

**FIGURE 1:**
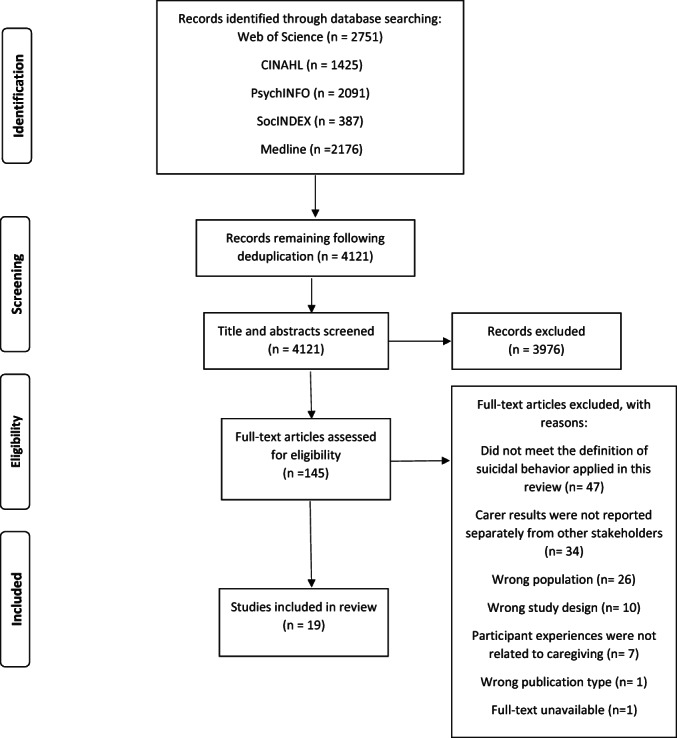
PRISMA flow diagram of study selection

Figure 1 presents the process of study identification. Database searches returned 4121 unique articles. Of these, 145 were read in full and checked against eligibility criteria. Nineteen studies published between 1987 and 2022 met eligibility for inclusion. Citation tracking returned no additional relevant articles. Characteristics of included articles are presented in Table 1.

**TABLE 1:** Characteristics of included studies

Authors (s) (year)	Aim/objective	Country	Description of carers	Description of persons receiving care	Data collection	Analytic approach
Asare‐Doku et al. (2017)	‘to understand the experiences of the families of [suicide] attempt survivors and how they cope with the aftermath of the attempt’	Ghana	10 family members (four fathers, two mothers, one brother, one husband, one aunt and one sister)	10 suicide attempt survivors who had received care at an emergency unit	Semi‐structured interviews	Interpretive Phenomenological Analysis
Buus et al. (2014)	‘to gain further insights into the experiences of parents of sons or daughters who have attempted suicide and how these parents respond to the increased psychosocial burden following the attempt(s)’	Denmark	14 parents (nine mothers, six fathers) attending a support programme for relatives of people who had attempted suicide	Suicide attempt survivors (14–35 years)	Focus groups	Thematic analysis
Daly (2005)	‘describes and enhances the understanding of what life is like for six mothers living with suicidal adolescents’	Canada	Six mothers participating in outpatient family therapy	Adolescents (12–16 years) ‘diagnosed with mental illness…and had exhibited suicidal behaviours’	Unstructured interviews	Phenomenological approach
Doyle et al. (2021)	‘to better understand Black mothers' and White mothers' qualitative reactions to their adolescents' hospitalizations due to suicide attempts’	United States	40 mothers (20 Black mothers and 20 White mothers) recruited approximately 1 month following a child's suicide attempt)	Adolescents (mean age 15 years) hospitalised due to suicide attempts	Semi‐structured interviews	Grounded theory
Dransart and Guerry (2017)	‘the study aimed at grasping how significant others perceived, were involved in, and dealt with suicidality…of loved ones, and what they did (or not) to seek help for their loved one or for themselves’	Switzerland	18 significant others (five partners, three children, three mothers, three sisters, two ex‐partners and two friends	19 people (19–77 years) assessed by their significant other to be suicidal or have attempted suicide	Semi‐structured interviews	Qualitative content analysis
Fu et al. (2021)	‘to explore parents' and the front‐line medical staff's experience of an adolescent with suicide‐related behaviours admitted to the psychiatry department of a general hospital in China’	China	15 parents (11 mothers, four fathers)	Adolescents (12–18 years) with suicide‐related behaviours (suicidal ideation, planning or attempts) receiving care at a psychiatric ward	Semi‐structured interviews	Thematic analysis
Garcia‐Williams and McGee (2016)	‘qualitatively describing the self‐reported responses college students have engaged in, at any point in their lifetime, when a friend or family member disclosed being suicidal’	United States	126 undergraduate students	Student‐identified family member or friend who had experienced suicidal thoughts	Open‐ended online survey	Thematic analysis
Hellerova et al. (2022)	‘to determine caregivers' perceptions about mental illness in their children, specifically regarding suicidality and depression, the impact of the children's mental health on the caregiver, and barriers to and facilitators of treatment’	United States	20 mothers (primary caregivers)	Children (6–17 years) presenting with suicidal behaviour to a paediatric emergency department	Semi‐structured interviews	Qualitative descriptive methodology
Inscoe et al. (2021)	‘The purpose of this study was to identify, from the perspective of caregivers, clinical practices that are sensitive to the needs of youth with co‐occurring traumatic stress and suicidal thoughts and behaviours, as well as common barriers to receiving care.’	United States	13 caregivers (12 female, one male)	Youth with caregiver‐identified histories of trauma and co‐morbid suicidal behaviour who had accessed community mental health services	Semi‐structured interviews	Grounded theory
de Lange et al. (2021)	‘to explore experiences and needs related to formal and informal mental healthcare for SGM [sexual and gender minority] youth who experience suicidal ideation’	Netherlands	16 parents (11 mothers, five fathers)	Sexual and gender minority youth (11–22 years) with parent‐identified histories of suicidal ideation	Semi‐structured interviews	Reflexive thematic analysis
Ngwane and van Der Wath (2019)	‘to understand the psychosocial support required by parents through exploring their lived experiences of how they made sense of their adolescents' attempted suicide’	South Africa	10 mothers	Children receiving inpatient psychiatric care following a suicide attempt	Semi‐structured interviews	Thematic analysis
Nosek (2008)	‘examined the process families use to care for a depressed and suicidal family member at home’	United States	17 family members (seven partners, one sibling, one daughter, and eight parents)	People with a recent episode of depression (within 6 months) who had been hospitalised due to suicide risk	Semi‐structured interviews	Grounded theory
Nygaard et al. (2019)	‘to gain insight into how a parent's relationship with a partner was affected after their son or daughter's suicide attempt or serious suicide threats’	Denmark	19 parents (seven fathers, 12 mothers) in contact with a counselling and support organisation for families affected by suicidal behaviour	People (14–54 years) who had either made suicide threats or attempted suicide	Semi‐structured interviews	Thematic analysis
Sellin et al. (2017)	‘to describe the phenomenon of participation, as experienced by relatives of persons who are subject to inpatient psychiatric care due to a risk of suicide’	Sweden	8 (five women, three men) ‘close relatives’	People who had received inpatient psychiatric care related to suicide risk	‘phenomenon‐oriented interviews’	Phenomenological approach
Thapa et al. (2021)	‘This study aims to find out the level of stress amongst caregivers of suicidal patients and various factors associated with it’	Nepal	Five primary caregivers (provided care for at least 6 months)	People who had attempted suicide and were receiving inpatient care	Semi‐structured interviews	Thematic analysis
Talseth et al. (2001)	‘to illuminate the meaning of relatives' lived experiences of being met by mental health care personnel during the care of their family member at risk of committing suicide’	Norway	15 ‘adult relatives’ (eight female, seven male)	15 people receiving inpatient psychiatric care at risk of suicide	Narrative interviews	Phenomenological hermeneutic method
Vandewalle et al. (2021)	‘to develop an understanding of family members' expectations of care and treatment for their [suicidal] relative’	Belgium	14 family members (five partners, three parents, three adult children, three siblings)	People receiving inpatient care who had experienced suicidal ideation in the previous year	Semi‐structured interviews	Grounded theory
Wolk‐Wasserman (1987a)	‘describe and analyse the abuse patients' and their significant others' efforts to obtain help from public care institutions in the presuicidal situation’	Sweden	33 significant others (13 partners, 10 parents, 10 other relatives or friends)	People admitted to an intensive care united because of intoxication with the purpose of attempting suicide	Semi‐structured interviews	Type of analysis not given
Wolk‐Wasserman (1987b)	‘to describe and analyse attempts by neurotic and prepsychotic/psychotic patients and their significant others to seek help from psychiatric, somatic and social care institutions in the presuicidal situation, and to analyse the reasons why the contacts with care institutions failed to have a preventative effect’	Sweden	37 significant others (11 partners, 13 parents, 13 other relatives or friends)	People ‘classified as neurotic or prepsychotic/psychotic’ admitted to an intensive care unit after attempting suicide by overdose and/or consumption of alcohol	Semi‐structured interviews	Type of analysis not given

Results of the study appraisal (CASP, 2018) are presented in Table 2. Across all studies, qualitative methods were appropriate in light of their stated research aims (CASP items 1 and 2). However, 12 failed to provide sufficient detail to inform judgements regarding whether the relationship between the authors and participants had been considered (CASP item 6), reducing confidence that these articles had critically considered the authors' roles in the research process. Moreover, six studies provided only limited detail regarding their analytic procedure (CASP item 8), limiting their methodological transparency to the extent that judgements regarding analytic rigour were not possible. Notwithstanding these limitations, all studies provided convincing justification of the scientific and/or practice‐based value of their research findings (CASP item 10).

**TABLE 2:** CASP Qualitative Checklist results

First author (year)	CASP question number^a^									
	1	2	3	4	5	6	7	8	9	10
Asare‐Doku et al. (2017)	✓	✓	✓	✓	✓	—	✓	✓	✓	✓
Buus et al. (2014)	✓	✓	✓	✓	✓	✓	✓	—	✓	✓
Daly (2005)	✓	✓	✓	✓	✓	—	✓	✓	✓	✓
Doyle et al. (2021)	✓	✓	✓	✓	✓	✓	✓	✓	✓	✓
Dransart and Guerry (2017)	✓	✓	✓	✓	✓	—	✓	✓	✓	✓
Fu et al. (2021)	✓	✓	✓	✓	✓	—	✓	—	✓	✓
Garcia‐Williams and McGee (2016)	✓	✓	✓	✓	✓	—	✓	—	✓	✓
Hellerova et al. (2022)	✓	✓	✓	✓	✓	✓	✓	✓	✓	✓
Inscoe et al. (2021)	✓	✓	✓	✓	✓	✓	✓	✓	✓	✓
de Lange et al. (2021)	✓	✓	✓	✓	✓	—	✓	✓	✓	✓
Ngwane and van Der Wath (2019)	✓	✓	✓	✓	✓	—	✓	✓	✓	✓
Nosek (2008)	✓	✓	✓	✓	✓	—	✓	✓	✓	✓
Nygaard et al. (2019)	✓	✓	✓	✓	✓	✓	✓	✓	✓	✓
Sellin et al. (2017)	✓	✓	✓	✓	✓	✓	✓	✓	✓	✓
Thapa et al. (2021)	✓	✓	✓	✓	✓	—	✓	✓	✓	✓
Talseth et al. (2001)	✓	✓	✓	✓	✓	—	✓	—	X	✓
Vandewalle et al. (2021)	✓	✓	✓	✓	✓	✓	✓	✓	✓	✓
Wolk‐Wasserman (1987a)	✓	✓	—	✓	✓	—	✓	—	✓	✓
Wolk‐Wasserman (1987b)	✓	✓	—	✓	✓	—	✓	—	✓	✓

^a^ Yes (✓) No (X) Cannot tell (–). CASP questions: (1) Was there a clear statement of the aims of the research? (2) Is a qualitative methodology appropriate? (3) Was the research design appropriate to address the aims of the research? (4) Was the recruitment strategy appropriate to the aims of the research? (5) Was the data collected in a way that addressed the research issue? (6) Has the relationship between researcher and participants been adequately considered? (7) Have ethical issues been taken into consideration? (8) Was the data analysis sufficiently rigorous? (9) Is there a clear statement of findings? (10) How valuable is the research? (this final question has been adapted to ‘is there a clear statement of the value of the research?’ in the above table).

**FIGURE 2:**
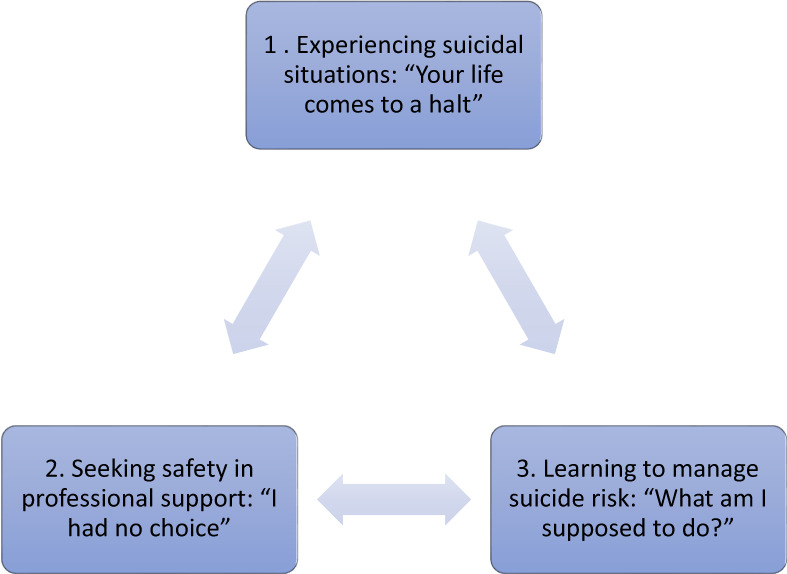
Analytic themes

Three interrelated analytic themes were developed (Figure 2), each with supporting descriptive themes. Table 3 presents the thematic framework alongside supporting illustrative quotes.

**TABLE 3:** Analytic themes (bolded) and descriptive themes (italicised) with illustrative quotes

Thematic framework	Illustrative quotes
1. Experiencing suicidal situations: ‘Your whole life comes to a halt’	‘If he would have been admitted to the right ward straightaway, he would never have never been so deeply affected… The 3‐week waiting period at home was a nightmare… it is like your life comes to a halt.… It is very serious for those with suicidal thoughts, but I dare to say that it is just as hard for the whole family to live in such a situation’ (Vandewalle et al., 2021, p. 1142)
*1.1 Emotional responses to suicidal behaviour: ‘I was completely floored’*	‘…it was very painful to me and I was thinking something that is not possible: reversing it and not seeing what has happened now’ (Ngwane & van Der Wath, 2019, p. 377)
*1.2 Coping alongside suicide risk*	‘I try not to let the fear overwhelm me…take it one day at a time’ (Doyle et al., 2021, p. 82)
2. Seeking safety in professional support: ‘I had no choice’	‘My only responsibility was to keep him alive. I strived to help him by going for walks, chatting… but that was not helping any longer. He was no longer safe with those suicidal thoughts. I had no choice other than hospitalisation’ (Vandewalle et al., 2021, p. 5)
*2.1 Sharing the responsibility of care*	‘I contacted their family members and described the severity of the situation so that [the suicidal peer] could get help in a hospital setting. They were very angry and felt I betrayed their trust, but I did it anyway because it was what they needed to prevent harm’ (Garcia‐Williams & McGee, 2016, p. 83)
*2.2 Being informed: ‘I feel safer at once’*	‘We were called to several meetings. We were told how things were and they informed us about the patient. They told us how they worked with (the patient) and that it would take time. We were informed about what has been happening the whole time. Now I understand my son's condition better’ (Talseth et al., 2001, p. 251)
*2.3 Being overlooked: ‘I never felt truly heard’*	‘I hope they understand that her situation is very critical. I expect that they keep her safe and have a treatment plan, which I can follow. But I am actually very uncertain about all that. They do not involve me, and I do not know what is happening’ (Vandewalle et al., 2021, p. 4)
*2.4 Barriers to accessing professional support*	‘On the day following his suicide attempt, I told myself ‘I really have to find a psychologist or someone’, well, I tried calling some and I was told everywhere ‘there is a 6‐month waiting list’ (Dransart & Guerry, 2017, p. 5)
3. Learning to manage risk: “what am I supposed to do?”	“I think it is my responsibility to show her the correct way, but if she does this [attempt suicide], what am I supposed to do?’ (Ngwane & van Der Wath, 2019, p. 379)
*3.1 Seeking understanding of suicidal behaviour*	‘I asked myself that maybe she had long standing problems and not knowing with whom to share with. I keep asking myself who made her sad, is it me or what, l kept asking myself questions. Initially if I failed somewhere, she could tell me that I have disappointed her or send me an SMS’ (Ngwane & van Der Wath, 2019, p. 377)
*3.2 Monitoring for risk: ‘It was red alert 24 hours a day’*	‘We'd go on this watch of when's it gonna happen?…we started to know him like the back of our hands. We knew exactly what he was doing…So you knew what the pattern was…but you did not know exactly when it was going to happen’ (Nosek, 2008, p. 40)
*3.3 The value of companionship*	‘…let [them] talk to me about what [they were] feeling and going through because [they] felt like [they] had no one to go to and I reassured [them] that [they] could always come to me’ (Garcia‐Williams & McGee, 2016, p. 82)

### Analytic theme 1: Experiencing suicidal situations: ‘your whole life comes to a halt’

Carers' experienced profound distress emerging from ongoing and pervasive fear of suicidal behaviour. Many carers consequently experienced significant challenges with coping and/or sought professional support to manage their distress.

### Descriptive theme 1.1: Emotional responses to suicidal behaviour: ‘I was completely floored’

Fourteen studies described participants' emotional reactions (Asare‐Doku et al., 2017; Buus et al., 2014; Daly, 2005; Doyle et al., 2021; Dransart & Guerry, 2017; Garcia‐Williams & McGee, 2016; Hellerova et al., 2022; Ngwane & van Der Wath, 2019; Nosek, 2008; Nygaard et al., 2019; Talseth et al., 2001; Thapa et al., 2021; Vandewalle et al., 2021; Wolk‐Wasserman, 1987b). The period following suicidal crises was described as ‘emotional turmoil’ (Daly, 2005, p. 27), leaving carers with shock and anger at the desire of their friend or family member to end their lives (Asare‐Doku et al., 2017; Buus et al., 2014; Daly, 2005; Doyle et al., 2021). Emphasising the magnitude of this emotional impact, one study identified post‐traumatic reactions to witnessing a suicide attempt, including re‐living the incident and emotional numbing (Ngwane & van Der Wath, 2019). Initial reactions gave way to ongoing stress as carers attempted to adapt to living alongside suicide risk (Buus et al., 2014; Daly, 2005; Doyle et al., 2021; Dransart & Guerry, 2017; Ngwane & van Der Wath, 2019; Nosek, 2008; Nygaard et al., 2019; Sellin et al., 2017; Talseth et al., 2001; Thapa et al., 2021; Vandewalle et al., 2021). Carers' persistent apprehension was described as ‘a constant concern. A constant worry…it's horrible’ (Nosek, 2008, p. 39), with one participant recalling ‘you can't sleep properly at night and you sit around feeling anxious all day. It's a dreadful situation to be in’ (Talseth et al., 2001, p. 253).

Context‐specific factors influenced carers' emotional reactions. This included social stigma, with some carers marginalised by other family and community members (Asare‐Doku et al., 2017; Daly, 2005; Ngwane & van Der Wath, 2019; Wolk‐Wasserman, 1987b) and guilt focused on carers' perceived failures to prevent suicidal behaviour (Buus et al., 2014; Daly, 2005; Doyle et al., 2021; Ngwane & van Der Wath, 2019; Vandewalle et al., 2021; Wolk‐Wasserman, 1987b). Guilt was particularly prominent in parents, whose responsibility to protect their child was undermined by their suicidal behaviour (Buus et al., 2014; Daly, 2005; Doyle et al., 2021; Ngwane & van Der Wath, 2019).

### Descriptive theme 1.2: Coping alongside suicide risk

Fourteen studies communicated carers' coping needs and experiences. Coping strategies included trying to adopt an attitude of acceptance (Doyle et al., 2021; Ngwane & van Der Wath, 2019; Sellin et al., 2017; Talseth et al., 2001), drawing on social support (Asare‐Doku et al., 2017; Doyle et al., 2021; Hellerova et al., 2022; Ngwane & van Der Wath, 2019) and seeking spiritual guidance (Asare‐Doku et al., 2017; Doyle et al., 2021; Ngwane & van Der Wath, 2019). For others, time away from their caregiving responsibilities was necessary due to the highly demanding and unrelenting interpersonal context of caring for someone at risk of suicide (Doyle et al., 2021; Dransart & Guerry, 2017; Nosek, 2008). Indeed, thinking about and addressing a family member's experiences of suicidal behaviour was highly distressing (Asare‐Doku et al., 2017; Doyle et al., 2021; Ngwane & van Der Wath, 2019; Nosek, 2008), such that for some carers, avoidance and distraction became key coping strategies: ‘I keep myself busy… because I don't want to think … I could have been burying my child’. (Doyle et al., 2021, p. 83). Two studies framed coping with respect to socio‐cultural factors. A US study reported that both black and white mothers drew on prayer and social support, while white women focused more specifically on their experiences of distress and the need for professional support (Doyle et al., 2021). A study conducted in Ghana (Asare‐Doku et al., 2017) identified social support as a coping resource for mothers, but not fathers, interpreted by the author as evidence of gendered responses to suicidal behaviour within the family.

Some carers had sought professional emotional support, primarily in the form of individual counselling (Garcia‐Williams & McGee, 2016; Nosek, 2008; Nygaard et al., 2019; Vandewalle et al., 2021; Wolk‐Wasserman, 1987a) while others identified personal professional emotional support as an unresolved support need (Dransart & Guerry, 2017; Inscoe et al., 2021; Ngwane & van Der Wath, 2019; Wolk‐Wasserman, 1987b).

### Analytic theme 2: Seeking safety in professional support: ‘I had no choice’

Many carers sought support from professional healthcare services. Services that instilled a sense of competence and interpersonal openness were highly valued. Conversely, services that were hard to access, difficult to understand, and did not seek to include carers' own expertise in managing suicide risk exacerbated carer frustration and anxiety.

### Descriptive theme 2.1: Sharing the responsibility of care

Thirteen studies described the experience of contacting professionals to help manage suicidal behaviour (Buus et al., 2014; de Lange et al., 2021; Dransart & Guerry, 2017; Garcia‐Williams & McGee, 2016; Hellerova et al., 2022; Inscoe et al., 2021; Ngwane & van Der Wath, 2019; Nosek, 2008; Sellin et al., 2017; Talseth et al., 2001; Vandewalle et al., 2021; Wolk‐Wasserman, 1987a, 1987b). Carers primarily desired help to establish their friend or family member's physical safety when they felt unprepared to manage suicidal behaviour (Dransart & Guerry, 2017; Garcia‐Williams & McGee, 2016; Sellin et al., 2017; Vandewalle et al., 2021; Wolk‐Wasserman, 1987a). Sharing this heavy responsibility was a source of profound relief for some carers (Garcia‐Williams & McGee, 2016; Sellin et al., 2017; Talseth et al., 2001; Wolk‐Wasserman, 1987b). Indeed, regarding hospital admission, one carer noted: ‘I am always restless and anxious, at home, at work… and then his admission created a moment of rest, to take some time off for myself. Because I knew: they will take care of him’ (Vandewalle et al., 2021, p. 1142).

### Descriptive theme 2.2: Being informed: ‘I feel calmer at once’

Eight studies described the nature and value of effective communication with healthcare professionals (de Lange et al., 2021; Dransart & Guerry, 2017; Garcia‐Williams & McGee, 2016; Inscoe et al., 2021; Sellin et al., 2017; Talseth et al., 2001; Thapa et al., 2021; Vandewalle et al., 2021). Carers valued proactive information sharing: ‘…in the evening, at 19h, the psychiatrist calls me and then she tells me “you know, I have contacted your husband's GP…”. I found this fantastic!’ (Dransart & Guerry, 2017, p. 7) and appreciated a collaborative interpersonal approach (Inscoe et al., 2021; Sellin et al., 2017; Vandewalle et al., 2021; Wolk‐Wasserman, 1987b) characterised by a validating attitude and reassurance: ‘It felt good for somebody to look at me as a mother and say, ‘Hey mom, you're hurting too. And that's okay’ (Inscoe et al., 2021, p. 656). Exemplifying the significance of professionals’ interpersonal styles, one participant identified that:

That therapist says the same things as others, but in a way that makes me feel, ‘Okay I know I am falling short on this, but I can improve’. I like that style. When I speak to this other [Mental Health Professionals] it is like ‘You are falling short’, which makes me feel guilty. (Vandewalle et al., 2021, p. 1144)

### Descriptive theme 2.3: Being overlooked: ‘I never felt truly heard’

Ten studies detailed carers' difficulties communicating effectively with healthcare professionals (Dransart & Guerry, 2017; Fu et al., 2021; Inscoe et al., 2021; Ngwane & van Der Wath, 2019; Nosek, 2008; Sellin et al., 2017; Talseth et al., 2001; Vandewalle et al., 2021; Wolk‐Wasserman, 1987a, 1987b). Lack of information provision left carers disempowered (Dransart & Guerry, 2017; Talseth et al., 2001; Vandewalle et al., 2021), underinformed about their friend or family member's care (Dransart & Guerry, 2017; Talseth et al., 2001) and unsure whether professionals had a thorough understanding of the severity of the situation (Dransart & Guerry, 2017; Fu et al., 2021; Talseth et al., 2001). Reflecting on their experience of attending hospital, one carer recalled: ‘A person is sitting there who you know you cannot leave for half an hour because she will try to take her own life, and no one listens to you…’ (Talseth et al., 2001, p. 253). Other consequences of limited communication with health professionals included feelings of guilt about the inadequacy of carers' own actions (Ngwane & van Der Wath, 2019; Vandewalle et al., 2021) and a sense of being an informant rather than a partner in care (Dransart & Guerry, 2017; Vandewalle et al., 2021; Wolk‐Wasserman, 1987b). One carer described this as ‘a feeling of not being listened to, of not knowing where to go, whom to reach out to, how to find help…we are alone, powerless, we don't know what to do’ (Dransart & Guerry, 2017, p. 6). Some carers were dejected by the inability of staff to factor their understanding of the situation into the care offered to service users (Dransart & Guerry, 2017; Fu et al., 2021; Sellin et al., 2017; Talseth et al., 2001; Vandewalle et al., 2021; Wolk‐Wasserman, 1987a):

The real disappointment for me was when her suicide attempt led her to the hospital, but after three days, they just released her and that was it. Yet I told them ‘but listen, she is not ready to get out, we've been dealing with this for ten years, you can be sure that she will try again’. (Dransart & Guerry, 2017, p. 6).

### Descriptive theme 2.4: Barriers to accessing professional support

Ten studies highlighted additional barriers to professional support (Buus et al., 2014; Daly, 2005; Dransart & Guerry, 2017; Fu et al., 2021; Hellerova et al., 2022; Inscoe et al., 2021; Talseth et al., 2001; Thapa et al., 2021; Vandewalle et al., 2021; Wolk‐Wasserman, 1987a). This included long waiting times and a lack of hospital bed availability (Dransart & Guerry, 2017; Fu et al., 2021; Hellerova et al., 2022; Talseth et al., 2001; Vandewalle et al., 2021). Others described how treatment options were insufficient to meet the needs of a family affected by suicidal behaviour, due to staff misunderstanding the nature of the situation (de Lange et al., 2021; Wolk‐Wasserman, 1987a) or because services were focused to a greater degree on physical health (Fu et al., 2021). Other barriers included lack of continuity in care, causing carers to repeat distressing details to multiple staff members (Dransart & Guerry, 2017), an absence of effective follow‐up care (Vandewalle et al., 2021) and the prohibitively expensive cost of private healthcare (Garcia‐Williams & McGee, 2016; Hellerova et al., 2022; Thapa et al., 2021).

### Analytic theme 3: Learning to manage risk: ‘what am I supposed to do?’

Carers struggled with making sense of the reasons for suicidal behaviour and therefore how to intervene to prevent its reoccurrence. A reflexive and emotionally exhausting monitoring of friends' and family members' emotional and physical states was a common response to this situation, especially in carers' early experiences.

### Descriptive theme 3.1: Seeking to understand suicidal behaviour

Participants in 14 studies reflected on the process of developing a deeper understanding of suicidal behaviour and their role in its management (Daly, 2005; de Lange et al., 2021; Doyle et al., 2021; Dransart & Guerry, 2017; Fu et al., 2021; Garcia‐Williams & McGee, 2016; Ngwane & van Der Wath, 2019; Nosek, 2008; Nygaard et al., 2019; Sellin et al., 2017; Talseth et al., 2001; Thapa et al., 2021; Vandewalle et al., 2021; Wolk‐Wasserman, 1987a). Initially, some carers experienced a state of being ‘thrown into it’ (Vandewalle et al., 2021, p. 1143), with a disorientating lack of understanding of what to do informing a sense of ‘having blindfolds on’ or ‘being in the dark’ (Nosek, 2008, p. 39). Carers felt ill‐equipped to offer support (Dransart & Guerry, 2017; Nygaard et al., 2019; Vandewalle et al., 2021), powerless regarding how to help (Ngwane & van Der Wath, 2019; Talseth et al., 2001), and unsure how to behave around someone experiencing suicidal behaviour (Daly, 2005; Ngwane & van Der Wath, 2019). One study described understanding suicidal behaviour as a cyclical process, involving observing a family member, intervening to help, and revising their supportive approach: ‘OK, let's not try that again, let's try this approach…and a lot of it was hit and miss’ (Nosek, 2008, p. 40). This iterative process of experiential learning helped carers who were aware of suicidal behaviour ‘to take the second step…being able to cope with the behaviour’ (Nosek, 2008, p. 40). Nine studies highlighted professional assistance with interpersonal support strategies as a key support need (de Lange et al., 2021; Doyle et al., 2021; Dransart & Guerry, 2017; Fu et al., 2021; Garcia‐Williams & McGee, 2016; Nosek, 2008; Vandewalle et al., 2021; Wolk‐Wasserman, 1987a):

Educating all parties on what to look for, what's happening, you know that was helpful with us just really trying to know the signs because a lot of people just don't know what suicidal thoughts look like or what does this shift in behavior mean and the little nuances that a kid might go through that is having those thoughts. (Inscoe et al., 2021, p. 656).

### Descriptive theme 3.2: Monitoring risk: ‘It was red alert 24 hours a day’

Carers in 13 studies reflected on their heightened degree of vigilance to and monitoring of suicidal behaviour amongst their friends or family members (Buus et al., 2014; Daly, 2005; Doyle et al., 2021; Dransart & Guerry, 2017; Garcia‐Williams & McGee, 2016; Hellerova et al., 2022; Ngwane & van Der Wath, 2019; Nosek, 2008; Nygaard et al., 2019; Sellin et al., 2017; Talseth et al., 2001; Thapa et al., 2021; Vandewalle et al., 2021). This heightened state of awareness was a response to ongoing anxieties around potential suicidal behaviour, mitigated by increasing the frequency of contact with a friend or family member (Doyle et al., 2021; Ngwane & van Der Wath, 2019; Nosek, 2008; Sellin et al., 2017; Vandewalle et al., 2021). This resulted in carers feeling ‘“sleep deprived”, “exhausted”, “very tired all of the time”, or that they “can't have one peaceful night [sleep]”’ (Doyle et al., 2021, p. 81). In some circumstances, monitoring for means of self‐injury represented an important support strategy:

Let me describe a typical day. I tiptoe in her room and watch the clothes, looking at the blankets to see if they are moving up and down. Then I check the pill bottles. I give her the ones she needs, and then I count what is left. Depending on what kind of workday I'm having, I'll probably go home at lunch to check on her. Sometimes I take all the pills with me to work in my purse. (Daly, 2005, p. 27).

### Descriptive theme 3.3: The value of companionship

Nine studies described the therapeutic value of companionship (Buus et al., 2014; de Lange et al., 2021; Doyle et al., 2021; Dransart & Guerry, 2017; Garcia‐Williams & McGee, 2016; Ngwane & van Der Wath, 2019; Nosek, 2008; Nygaard et al., 2019; Vandewalle et al., 2021). Participants described the importance of making themselves available for their loved one in any way they found useful (Sellin et al., 2017), providing moral support (Dransart & Guerry, 2017), and convincing their suicidal friend or family member of their value to others (Garcia‐Williams & McGee, 2016). As one participant stated, simply being with their family member was perceived to be beneficial:

it gives a lot when the family manages it and when you bear to be close, and you bear to be there. Thus, it is enough just to sit and watch TV together, just such a thing. Just that you are demonstrating that I am here. So that means probably a lot I believe (Sellin et al., 2017, p. 4).

Companionship also involved engaging family members or friends in everyday activities to ‘keep their mind off the [depression]’ (Nosek, 2008, p. 41); supporting them with accessing health services (Garcia‐Williams & McGee, 2016; Nosek, 2008); and discuss their mental health experiences (de Lange et al., 2021; Ngwane & van Der Wath, 2019; Nygaard et al., 2019; Sellin et al., 2017; Vandewalle et al., 2021), often in a compassionate and supportive manner: ‘I sat down and talked them through it. I told them how much I cared…’ (Garcia‐Williams & McGee, 2016, p. 82).

## DISCUSSION

This review synthesised 19 qualitative studies investigating carers' experiences of supporting friends or family members with suicidal behaviour. Carers' emotional reactions to suicidal behaviour were characterised by stress and anxiety emerging from the possibility of suicide. Carers attempted to understand and manage the ongoing uncertainty of living alongside suicidal behaviour by adopting strategies including monitoring for signs of suicide risk and offering compassionate emotional support. Yet while carers frequently sought support from safety‐focused and carer‐inclusive health services, access to such support was often limited.

This qualitative review contextualises quantitative evidence of increased caregiving burden (McDonell et al., 2003) and poorer well‐being (Morgan et al., 2013) amongst carers of people who have experienced suicidal behaviour. For carers, recovery is likely to be highly idiosyncratic and influenced by factors such as the availability of professional support and sociocultural perceptions of suicidal behaviour and caregiving. Yet across contexts, results here suggest that recovery is likely to be facilitated by improving carer self‐efficacy in reducing suicide risk. As in prior research, carers require support with developing interpersonal support strategies and with re‐establishing positive relationships within families (Sun et al., 2008, 2009; Sun & Long, 2013). This review suggests that meeting these needs may help to mitigate the disempowering and anxiety‐inducing response to the possibility of future suicidal behaviour, which contributes to an emotionally fatiguing reliance on hypervigilance amongst carers.

Previous qualitative reviews have characteristically included carers of people experiencing self‐injury regardless of intent, and/or recruited family carers, particularly parents (Juel et al., 2020; Lachal et al., 2015; Simes et al., 2021). Results reported here suggest that disempowerment, fear, and a pressing need to keep a loved one safe, are relatively consistent carer experiences across this literature. Building on this prior evidence, this review suggests that these challenges are likely to be influenced by the extent to which carers feel able to meet their personal responsibility for protecting those they support from suicide. Carer distress, particularly guilt experienced by parents, was most prominent where they perceived that this responsibility was undermined. Whether this varies by relationship type is unclear. The broad carer‐related inclusion criterion used here revealed that a majority of research in this area relates to family experiences. This limitation of existing literature is significant given that friends are a key source of support for people experiencing suicidal behaviour (Czyz et al., 2012; Giletta et al., 2017; Massing‐Schaffer et al., 2020). Further research investigating the significance of relationship type and caregiving in the context of suicidal behaviour is necessary to elucidate the support needs of those in this important yet under investigated caregiving role.

Carers experience significant distress in periods immediately following suicidal behaviour and on a more enduring basis. This aligns with what Buus et al. (2014) describe as ‘double‐crisis’, one experienced at the point of suicidal behaviour and the other reflecting the ongoing impact of trauma and stress on families. Several potential models of intervention involving are relevant to these circumstances. Family Intervention for Suicide Prevention is a cognitive behavioural approach including initial emergency care for suicidal service users and ongoing family support, which is effective in increasing follow‐up care utilisation (Hughes & Asarnow, 2013). Longer term psychotherapeutic approaches are also relevant, with the strongest evidence for dialectical behaviour therapy for young people experiencing self‐injury and their families (Glenn et al., 2019). However, this review suggests that carers face difficulties accessing such support and the extent to which similar resources have been successfully upscaled into clinical practice is unclear.

Carer support needs highlighted by this review, such as timely access to information about how to support someone at risk of suicide, make relevant methods of support that are accessible and scalable. Evidence‐based online resources tailored for this context hold the potential to meet these needs, the development of which has been spearheaded by charitable organisations. One example is the ‘You Are Not Alone’ online resource hosted by SANE Australia, informed by qualitative research with carers of people affected by suicidal behaviour (Coker et al., 2019). This resource includes information about what to do in crises and emotional support for carers, issues that relate directly to those identified by this review. Other similar resources such as the online toolkit for people impacted by suicide attempts (Mental Health Commission of Canada, 2018) and guidance for parents of young people who self‐harm hosted by the UK platform HealthTalk (Ferrey et al., 2016) represent sources of evidence‐informed psychoeducation for carers directly relevant to this context. Further research investigating the cross‐cultural applicability, effectiveness, and implementation of such resources is warranted.

For carers, positive experiences of health services were characterised by collaborative, safety‐focused relationships with professionals, and, conversely, negative ones by poor communication. Efforts should therefore be made to improve training and support for healthcare professionals likely to interact with carers of people at risk of suicide. Indeed, general practitioners have reported the desire for professional education in suicide prevention (Michail & Tait, 2016) and psychiatric inpatient staff have identified training needs related to developing therapeutic relationships with suicidal service users (Awenat et al., 2017). Relevant models of intervention delivered by or to healthcare professionals have been evaluated that may address these needs. For example, specialised emergency room care combining staff training and family therapy has been demonstrated to reduce suicidal behaviour (Rotheram‐Borus et al., 2000) and nurse‐delivered family psychoeducation focused on suicide prevention improves perceived caring ability amongst family carers (Sun et al., 2014). Involving professionals in suicide prevention training with a focus on the role of carers is therefore feasible and, as per the results of this review, should be an important consideration in the design of health services.

### Implications for practice and research

Carers require tailored support with how to care for a friend or family member at risk of suicide. Practitioners may seek to draw on evidence‐based strategies in pursuit of this goal, for example, by involving carers in crisis or safety planning (Stanley & Brown, 2012) but also by building collaborative interpersonal relationships with carers. Research with carers who are in contact with mental health services following suicidal situations has highlighted how professional‐service user confidentiality can confound the development of such relationships (Dransart & Guerry, 2017; Nosek, 2008). In order to balance carer involvement, service user safety, and the obligation to maintain service user confidentiality, practitioners could consult dedicated, evidence‐informed guidelines for determining how and when to disclose risk‐related information with carers (National Suicide Prevention Alliance, 2021).

Carers also require support with managing their own well‐being. To date, some psychosocial interventions have demonstrated positive impacts on carer outcomes including reduced caregiver burden and improved caregiving self‐efficacy (Krysinska et al., 2021). The findings of this review reinforce the recommendations made by Krysinska and colleagues (Krysinska et al., 2021) for the need to upscale and evaluate interventions for carers in order to provide practitioners with evidence‐based models of support. Empirical and theoretical research supporting the significance of social factors in suicidal behaviour could inform intervention development and meet carers needs for accessible interpersonal support strategies (Mueller et al., 2021). For example, targeting specific mechanisms implicated in suicidal behaviour, such as thwarted belongingness and perceived burdensomeness (O'Connor & Kirtley, 2018), has been shown to be feasible as part of clinician‐delivered interventions (Allan et al., 2018; Short et al., 2019). Future research may seek to combine this knowledge with developments in related domains, including communication and support skills training for carers of people with physical and mental health difficulties (Morris et al., 2018; Treasure & Todd, 2016), and could capitalise on growing research into the use of digital health platforms to deliver carer‐directed psychoeducation and peer support (Lobban et al., 2017; Sin et al., 2022) to overcome service access barriers.

### Strengths and limitations

This qualitative synthesis represents a novel contribution to the literature by applying qualitative synthesis to primary data derived from studies of caring experiences related to suicidal behaviour only and by extending the definition of carer to include friends. However, the scope of this review was limited to English language studies, owing to limitations in access to translation services. The transferability of these findings to other contexts captured by non‐English literature is limited. Including only published studies ensured that they had met the standard for publication, but may have excluded relevant qualitative data within grey literature.

## CONCLUSION

There is a pressing need to understand the social context of suicidal behaviour to inform suicide prevention strategies worldwide. Yet, as the results of this review demonstrate, the lived experiences of caring for a friend or family member who is or has been suicidal are characterised by significant emotional and interpersonal challenges, in addition to difficulties accessing appropriate professional support. This evidence justifies a renewed research focus on psychosocial support for carers.

## AUTHOR CONTRIBUTIONS

**Paul Marshall:** Conceptualization; formal analysis; methodology; project administration; writing – original draft; writing – review and editing. **Keith Sansom:** Formal analysis; writing – review and editing. **Glorianna Jagfeld:** Methodology; project administration; writing – review and editing. **Steven Jones:** Conceptualization; methodology; supervision; writing – review and editing. **Fiona Lobban:** Conceptualization; formal analysis; methodology; supervision; writing – review and editing.

## CONFLICT OF INTEREST

All authors declare no conflict of interest.

## Data Availability

Data sharing is not applicable to this article as no new data were created or analysed in this study.
